# Blood pressure control and treatment status at 1 year after the first health check-up in individuals with observed referral-level blood pressure

**DOI:** 10.1038/s41440-025-02284-y

**Published:** 2025-07-25

**Authors:** Kaori Kitaoka, Hidehiro Kaneko, Yuta Suzuki, Akira Okada, Atsushi Mizuno, Katsuhito Fujiu, Norifumi Takeda, Hiroyuki Morita, Tatsuhiko Azegami, Kaori Hayashi, Koichi Node, Yuji Furui, Katsuyuki Miura, Hideo Yasunaga, Norihiko Takeda

**Affiliations:** 1 NCD Epidemiology Research Center, Shiga University of Medical Science, Otsu, Japan; 2 Department of Cardiovascular Medicine, The University of Tokyo, Tokyo, Japan; 3 Department of Advanced Cardiology, The University of Tokyo, Tokyo, Japan; 4 Center for Outcomes Research and Economic Evaluation for Health, National Institute of Public Health, Saitama, Japan; 5 Department of Prevention of Diabetes and Lifestyle-Related Diseases, Graduate School of Medicine, The University of Tokyo, Tokyo, Japan; 6 Department of Cardiovascular Medicine, St. Luke’s International Hospital, Tokyo, Japan; 7 Division of Nephrology, Endocrinology and Metabolism, Department of Internal Medicine, Keio University School of Medicine, Tokyo, Japan; 8 Department of Cardiovascular Medicine, Saga University, Saga, Japan; 9 Healthcare Data Science Research Unit, Institute for Future Initiatives, The University of Tokyo, Tokyo, Japan; 10 Department of Public Health, Shiga University of Medical Science, Otsu, Japan; 11 Department of Clinical Epidemiology and Health Economics, School of Public Health, The University of Tokyo, Tokyo, Japan

**Keywords:** Hypertension, Blood pressure management, Health check-up, Obesity, Lifestyle factors.

## Abstract

Hypertension is a major risk factor for cardiovascular diseases. This study aimed to clarify blood pressure (BP) management after the health check-up among individuals with grade II or severer hypertension, defined as systolic BP (SBP) ≥ 160 mmHg or diastolic BP (DBP) ≥ 100 mmHg in Japan. This retrospective study used the JMDC Claims Database (2005–2022) in Japan, including 63,785 individuals (median age 50 years; men 75.3%) with BP above grade II hypertension thresholds during the health check-up. We evaluated the BP control and treatment status at 1 year after the first check-up. Poisson regression with robust error variance analyses were performed to assess the association with grade II or severer hypertension at 1 year after the first check-up. Notably, 45.4% continued to have grade II or severer hypertension at 1 year after the first check-up. Among the individuals, 54.5% visited a medical institution within 3 months after undergoing a health check-up, only 23.6% were prescribed antihypertensive medications at 1 year after the first check-up. Factors associated with sustained grade II or severer hypertension included per 5 years lower in age (relative risk [RR]: 1.01, 95% confidence interval [CI]: 1.01–1.02), obesity (RR: 1.04, 95% CI: 1.02–1.06), and skipping breakfast ≥3 times per week (RR: 1.06, 95% CI: 1.04–1.08). Despite strong recommendations for medical consultation, BP control remains inadequate among individuals with grade II or severer hypertension, nearly half of individuals had SBP ≥ 160 mmHg or DBP ≥ 100 mmHg at 1 year after the first check-up in our study. The findings highlight the need for prompt follow-up, particularly among younger adults and those with unhealthy lifestyles.

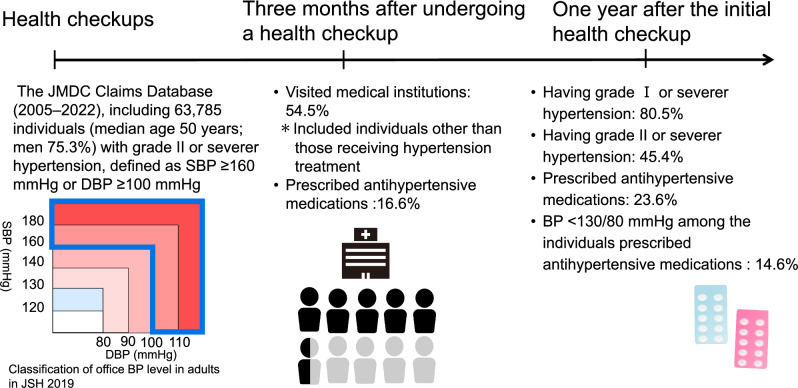

## Introduction

Hypertension is the most prevalent risk factor for cardiovascular disease (CVD) and has a large impact on cardiovascular outcomes [1–3]. Managing and controlling hypertension is crucial for preventing subsequent CVD. The global prevalence of hypertension is increasing due to population aging and greater exposure to lifestyle risk factors, such as unhealthy diets and physical inactivity [4]. The proportions of hypertension awareness, treatment, and control that have been reported across many countries vary substantially. Over the past three decades, the proportion of hypertension patients with controlled blood pressure (BP) has risen globally, particularly in high-income Asia-Pacific nations, except Japan [3, 5]. According to a 2016 Japanese survey, the proportion of patients with controlled BP—defined as systolic BP (SBP) < 140 mmHg and diastolic BP (DBP) < 90 mmHg—ranged from 33.3% to 48.0% [6, 7].

The Industrial Safety and Health Act mandates that employers must ensure that their employees undergo annual medical examinations conducted by a physician in Japan. In addition, Specific Health Check-ups and Specific Health Guidance are unique Japanese healthcare initiatives aged from 40 to 74 years aimed at the early detection and prevention of lifestyle-related diseases, focusing on metabolic syndrome through health examinations and guidance to reduce the risk of disease onset, particularly CVD [8, 9]. It is recommended that individuals with SBP ≥ 160 mmHg or DBP ≥ 100 mmHg, as identified during the health check-ups, seek medical consultation promptly [6, 8]. However, there has been no detailed analysis of how individuals with SBP ≥ 160 mmHg or DBP ≥ 100 mmHg during health check-up are treated, and how their BP control evolves. This study aimed to clarify the BP control and treatment status in individuals in whom referral-level BP was observed at the health check-up, using a large-scale Japanese health check-up and claims database.

Point of view

- Clinical relevance:
  This study highlights a critical treatment gap in individuals with grade II or more severe hypertension identified through health check-ups, as those who were younger, obese, or had unhealthy lifestyles did not initiate antihypertensive therapy despite recommendations.
- Future direction:
  There is a critical need to establish structured follow-up strategies and implement interventions that enhance physician engagement and promote patient adherence in hypertension management.

## Methods

### Study design and data source

The current study was a retrospective observational analysis using data from the JMDC Claims Database (JMDC, Tokyo, Japan), which is a health claims database in Japan, between 2005 and 2022 [10–12]. The JMDC contracts with more than 60 insurers. Most insured individuals in the JMDC database are employees and dependents of relatively large Japanese companies. The JMDC Claims Database includes annual health check-up data, demographics, medical history, medications, and hospital claims with International Classification of Diseases, 10th Revision (ICD-10) coding.

**Fig. 1:**
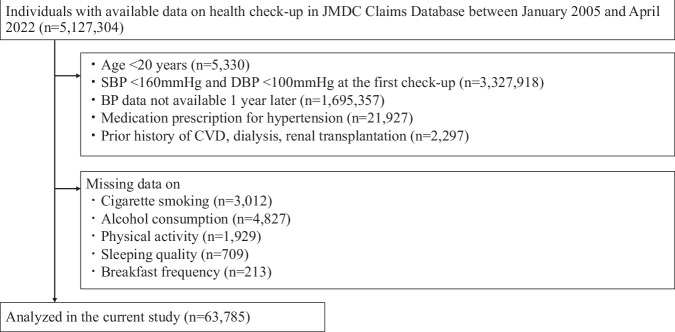
Flowchart of participant selection

Among the 5,127,304 individuals enrolled in the JMDC Claims Database, the current study focused on individuals aged 20–74 years who had SBP ≥ 160 mmHg or DBP ≥ 100 mmHg at the first health check-up and for more than 1 year after insurance enrollment (one- year look-back period). Exclusion criteria for the current study were as follows: (1) those aged <20 years (*n* = 5330), (2) those with SBP < 160 mmHg and DBP < 100 mmHg at the first check-up (*n* = 3,327,918), (3) those with BP data not available at 1 year after the first check-up (*n* = 1,695,357), (4) those with medication prescriptions for hypertension (*n* = 21,927), (5) those with a CVD history of myocardial infarction, angina pectoris, stroke, heart failure, dialysis, or renal transplantation (*n* = 2297), and (6) those with missing data on cigarette smoking (*n* = 3012), alcohol consumption (*n* = 4827), physical activity (*n* = 1929), sleeping quality (*n* = 709), and breakfast frequency (*n* = 213). Consequently, the current analysis included 63,785 participants. (Fig. 1).

The Japanese Ministry of Health, Labour and Welfare has provided a recommended protocol for BP measurement. According to the protocol, healthcare professionals were recommended to use either a standard sphygmomanometer or an automated device on the right arm, after the participants had been seated and at rest for 5 min. In principle, BP was recommended to be measured twice, and the average of the two readings was used for assessment, with a single measurement also considered acceptable depending on practical circumstances. When two measurements were performed, the mean of the first and second measurements was to be recorded. We obtained the medication prescriptions for hypertension (WHO-ATC codes starting with C02, C03, C04, C07, C08, or C09), dyslipidemia (WHO-ATC codes starting with C10), and hyperglycemia (WHO-ATC codes starting with A10), from the claims data after the health check-up. In the current study, the visit to a physician within 3 months was defined as any recorded outpatient visit to a medical facility within 3 months following the date of the first health check-up. For medication status, the prescription date listed in the claims data was used to determine the timing of antihypertensive medication use. The medication status at 1 year after the health check-up was determined based on the prescription data recorded on the date closest to the health check-up.

Obesity was defined as body mass index (BMI) ≥ 25 kg/m^2^. Diabetes was defined as fasting glucose ≥126 mg/dL or use of glucose-lowering medications. Dyslipidemia was defined as low-density lipoprotein cholesterol ≥140 mg/dL, high-density lipoprotein cholesterol <40 mg/dL, triglycerides ≥150 mg/dL, or use of lipid-lowering medications. During the health check-up, employees and dependents are required to complete a standardized self-reported questionnaire concerning their lifestyle habits. Information on cigarette smoking (current or noncurrent/never), alcohol consumption (every day or not every day), healthy sleep as good rest with sleep (yes or no), dietary habit as skipping breakfast ≥3 times per week (yes or no) was collected. We also defined physical inactivity as not performing 30 min of exercise at least twice a week or not walking for more than 1 h per day [13].

### Ethics

This study was approved by the University of Tokyo Ethics Committee (approval by the Institutional Review Board of the University of Tokyo: 2018–10862), and was conducted according to the Declaration of Helsinki. Because all data in the JMDC Claims Database were de-identified, the requirement for informed consent was waived in the current study.

### Statistical analysis

Descriptive statistics are presented as median (interquartile range (IQR)) and number (percentage). We compared individuals based on their visit to a physician within 3 months after the first health check-up and their antihypertensive treatment status at 1 year. In addition, we evaluated differences among BP categories for isolated grade II or severer systolic hypertension, isolated grade II or severer diastolic hypertension, and grade II or severer systolic and diastolic hypertension. Poisson regression with robust error variance analyses were performed to assess the association with SBP ≥ 160 mmHg or DBP ≥ 100 mmHg at 1 year after the first check-up [14, 15]. The relative risk (RR) was calculated after adjustment for potential confounders, including each of baseline variables. In addition, we performed an analysis adjusted for a variable of the visit to a physician within 3 months after undergoing a health check-up. Moreover, we performed analyses to evaluate independent factors associated with antihypertensive prescription.

Additionally, we compared baseline characteristics between individuals with and without follow-up data at 1 year after the first check-up. The latter group comprised only individuals who had available data on medical visits and antihypertensive medication prescriptions within 3 months after the first check-up. Moreover, these individuals had complete baseline information, including cigarette smoking, alcohol consumption, physical activity, sleeping quality, and breakfast frequency, resulting in 33,175 participants for analysis. Next, we compared individuals based on their visit to a physician within 3 months prior to the one-year follow-up health check-up and their antihypertensive treatment status at 1 year. We classified participants into the following categories based on their follow-up status: a) Follow-up BP ≥ 160/90 mmHg without medical visits, b) Follow-up BP < 160/90 mmHg without medical visits, c) Follow-up BP ≥ 160/90 mmHg with medical visits but without antihypertensive treatment, d) Follow-up BP < 160/90 mmHg with medical visits but without antihypertensive treatment, e) Follow-up BP ≥ 160/90 mmHg with medical visits and antihypertensive treatment, and f) Follow-up BP < 160/90 mmHg with medical visits and antihypertensive treatment. Also, we performed subgroup analyses according to age category, sex, presence or absence of obesity, and individuals aged 40–64 years. *P* values were calculated by the Mann-Whitney U test or the Kruskal-Wallis test for continuous variables, and chi-square tests for categorical variables. *P*-values <0.05 were used for statistical significance. All analyses were conducted using Stata version 18 software (StataCorp, College Station, TX, USA).

## Results

A total of 63,785 (men 75.3%) individuals were included in our real-world data (RWD) analysis. Among the study participants, 90.0% were insured employees. The median (IQR) age was 50 (44–56) years; median (IQR) SBP was 160 (149–167) mmHg; median (IQR) DBP was 103 (100–108) mmHg; and the median (IQR) BMI was 25.1 (22.6–28.3) kg/m^2^ at baseline (Table 1). Among those who were recommended to visit a medical institution, 34,789 individuals (54.5%) visited a physician within 3 months after undergoing a health check-up, and only 10,596 individuals (16.6%) were prescribed antihypertensive medications within 3 months after undergoing a health check-up. At 1 year after the first check-up, 28,930 individuals (45.4%) had grade II or severer hypertension and 51,321 individuals (80.5%) still had grade I or greater hypertension. 15,057 individuals (23.6%) were on antihypertensive medication at 1 year after the first check-up. Among the four subgroups, 11,266 (17.7%) visited a physician within 3 months and received antihypertensive treatment at 1 year after the first check-up, while 23,523 (36.9%) visited a physician but remained untreated. 3791 (5.9%) did not visit a physician within 3 months but were prescribed medication at 1 year after the first check-up, whereas 25,205 (39.5%) neither visited a physician nor received treatment. At 1 year after the first check-up, those who visited a physician within 3 months and received treatment had the lowest BP, with SBP at 136 (126–147) mmHg and DBP at 87 (80–94) mmHg. Although fewer in number, individuals who did not visit a physician within 3 months but were on antihypertensive medication at 1 year also had lower BP, with SBP at 137 (128–149) mmHg and DBP at 88 (82–96) mmHg. In contrast, individuals who did not receive treatment at 1 year after the first check-up had the higher BP.

**Table 1:** Comparison of individuals with and without visiting to a physician within 3 months after the first check-up and antihypertensive treatment at 1 year after the first check-up

Variables	Total (*n* = 63,785)	Visit to a physician within 3 months after the first check-up and with antihypertensive treatment (*n* = 11,266)	Visit to a physician within 3 months after the first check-up but without antihypertensive treatment (n = 23,523)	Not visit to a physician within 3 months after the first check-up but with antihypertensive treatment (*n* = 3791)	No visit to a physician within 3 months after the first check-up and without antihypertensive treatment (*n* = 25,205)	*P*-value
Age (years)	50 (44–56)	51 (45–56)	50 (44–56)	50 (45–55)	49 (43–55)	<0.001
Sex, men, n (%)	48,004 (75.3)	8,171 (72.5)	16,577 (70.5)	3,074 (81.1)	20,182 (80.1)	<0.001
Body mass index (kg/m^2^)	25.1 (22.6–28.3)	25.2 (22.8–28.3)	25.0 (22.4–28.1)	25.6 (23.2–28.7)	25.2 (22.6–28.4)	<0.001
Obesity, n (%)	33,008 (51.7)	5985 (53.1)	11,766 (50.0)	2,137 (56.4)	13,120 (52.1)	<0.001
Systolic blood pressure (mmHg)	160 (149–167)	162 (153–172)	158 (148–165)	162 (153–170)	160 (149–167)	<0.001
Diastolic blood pressure (mmHg)	103 (100–108)	104 (100–110)	102 (100–106)	104 (100–110)	103 (100–107)	<0.001
Diabetes mellitus, n (%)	5770 (9.0)	1111 (9.9)	2,361 (10.0)	349 (9.2)	1,949 (7.7)	<0.001
Dyslipidemia, n (%)	38,654 (60.6)	6855 (60.8)	14,313 (60.8)	2365 (62.4)	15,121 (60.0)	0.020
Cigarette smoking, n (%)	19,917 (31.2)	3305 (29.3)	5982 (25.4)	1417 (37.4)	9213 (36.6)	<0.001
Alcohol consumption, n (%)	23,491 (36.8)	4172 (37.0)	8092 (34.4)	1477 (39.0)	9750 (38.7)	<0.001
Physical inactivity, n (%)	35,523 (55.7)	6446 (57.2)	12,900 (54.8)	2270 (59.9)	13,907 (55.2)	<0.001
Poor sleep quality, n (%)	24,537 (38.5)	4584 (40.7)	9110 (38.7)	1507 (39.8)	9336 (37.0)	<0.001
Skipping breakfast ≥3 times per week, n (%)	17,687 (27.7)	2623 (23.3)	5519 (23.5)	1120 (29.5)	8425 (33.4)	<0.001
Fasting blood glucose (mg/dL)	98 (91–107)	98 (91–107)	98 (91–107)	98 (91–107)	98 (91–107)	0.073
Low-density lipoprotein-cholesterol (mg/dL)	132 (110–154)	131 (110–154)	131 (109–153)	134 (112–156)	133 (110–156)	<0.001
High-density lipoprotein-cholesterol(mg/dL)	58 (48–70)	58 (48–70)	59 (49–71)	56 (47–68)	57 (48–70)	<0.001
Triglycerides (mg/dL)	115 (78–173)	117 (80–174)	112 (76–169)	121 (81–179)	116 (79–175)	<0.001
Antihypertensive prescriptions within 3 months after undergoing a health checkup, n (%)	10,596 (16.6)	7992 (70.9)	1762 (7.5)	678 (17.9)	164 (0.7)	<0.001
Systolic blood pressure at 1 year after the first check-up (mmHg)	148 (137–161)	136 (126–147)	150 (140–162)	137 (128–149)	154 (142–166)	<0.001
Diastolic blood pressure at 1 year after the first check-up (mmHg)	96 (88–104)	87 (80–94)	97 (90–104)	88 (82–96)	99 (92–107)	<0.001

Data are expressed as median (interquartile range) or number (percentage). P values were calculated using the Kruskal-Wallis test for continuous variables and chi-square tests for categorical variables. We obtained the medication prescriptions for hypertension (WHO-ATC codes starting with C02, C03, C04, C07, C08, or C09), dyslipidemia (WHO-ATC codes starting with C10), and hyperglycemia (WHO-ATC codes starting with A10), from the claims data after the health checkup. Obesity was defined as body mass index ≥25 kg/m^2^. Diabetes was defined as fasting glucose ≥126 mg/dL or use of glucose-lowering medications. Dyslipidemia was defined as low-density lipoprotein cholesterol ≥140 mg/dL, high-density lipoprotein cholesterol <40 mg/dL, triglycerides ≥150 mg/dL, or use of lipid-lowering medications

Table 2 presented the baseline characteristics and treatment status of individuals categorized by hypertension subtype at their first health check-up. Those with isolated systolic hypertension were older and had a lower prevalence of obesity, whereas those with isolated diastolic hypertension had higher proportion of men, as well as higher prevalence of cigarette smoking and alcohol consumption. At 1 year after the first check-up, persistent grade II or severer hypertension was most frequent in individuals with grade II or severer systolic and diastolic hypertension (56.9%). The proportion of individuals receiving antihypertensive prescriptions at 1 year was also highest in this group (32.0%) and lowest among those with isolated diastolic hypertension (19.2%).

**Table 2:** Baseline characteristics and treatment status by hypertension subtypes at the first check-up

Variables	Isolated grade II or severer systolic hypertension (*n* = 13,081)	Isolated grade II or severer diastolic hypertension (*n* = 30,745)	Grade II or severer systolic and diastolic hypertension (*n* = 19,959)	*P*-value
Age (years)	55 (48–61)	48 (43–53)	50 (44–56)	<0.001
Sex, men, *n* (%)	7579 (57.9)	25,537 (83.1)	14,888 (74.6)	<0.001
Body mass index (kg/m^2^)	24.1 (21.7–27.2)	25.2 (22.8–28.2)	25.7 (23.0–29.1)	<0.001
Obesity, *n* (%)	5430 (41.5)	16,235 (52.8)	11,343 (56.8)	<0.001
Systolic blood pressure (mmHg)	164 (162–170)	149 (143–154)	169 (164–178)	<0.001
Diastolic blood pressure (mmHg)	93 (88–96)	103 (101–106)	108 (104–114)	<0.001
Diabetes mellitus, *n* (%)	1538 (11.8)	2055 (6.7)	2177 (10.9)	<0.001
Dyslipidemia, *n* (%)	7522 (57.5)	18,748 (61.0)	12,384 (62.0)	<0.001
Cigarette smoking, *n* (%)	3453 (26.4)	10,039 (32.7)	6425 (32.2)	<0.001
Alcohol consumption, *n* (%)	4409 (33.7)	11,759 (38.2)	7323 (36.7)	<0.001
Physical inactivity, *n* (%)	6784 (51.9)	17,484 (56.9)	11,255 (56.4)	<0.001
Poor sleep quality, *n* (%)	4706 (36.0)	12,097 (39.3)	7734 (38.7)	<0.001
Skipping breakfast ≥3 times per week, n (%)	2878 (22.0)	9005 (29.3)	5804 (29.1)	<0.001
Fasting blood glucose (mg/dL)	99 (91–109)	97 (90–105)	99 (91–109)	<0.001
Low-density lipoprotein-cholesterol (mg/dL)	131 (109–154)	131 (110–153)	133 (111–156)	<0.001
High-density lipoprotein-cholesterol(mg/dL)	62 (51–75)	56 (47–68)	57 (48–70)	<0.001
Triglycerides (mg/dL)	100 (70–152)	119 (81–179)	118 (81–179)	<0.001
Antihypertensive prescriptions within 3 months after undergoing a health checkup, n (%)	1907 (14.6)	3867 (12.6)	4822 (24.2)	<0.001
Grade II or severer hypertension at 1 year after the first check-up, *n* (%)	5065 (38.7)	12,500 (40.7)	11,365 (56.9)	<0.001
Antihypertensive prescriptions at 1 year after the first check-up, *n* (%)	2774 (21.2)	5897 (19.2)	6386 (32.0)	<0.001
Systolic blood pressure at 1 year after the first check-up (mmHg)	152 (140–164)	144 (134–154)	156 (141–170)	<0.001
Diastolic blood pressure at 1 year after the first check-up (mmHg)	90 (82–96)	97 (89–103)	99 (90–108)	<0.001

Data are expressed as median (interquartile range) or number (percentage). *P* values were calculated using the Kruskal-Wallis test for continuous variables and chi-square tests for categorical variables. We obtained the medication prescriptions for hypertension (WHO-ATC codes starting with C02, C03, C04, C07, C08, or C09), dyslipidemia (WHO-ATC codes starting with C10), and hyperglycemia (WHO-ATC codes starting with A10), from the claims data after the health checkup. Obesity was defined as body mass index ≥25 kg/m^2^. Diabetes was defined as fasting glucose ≥126 mg/dL or use of glucose-lowering medications. Dyslipidemia was defined as low-density lipoprotein cholesterol ≥140 mg/dL, high-density lipoprotein cholesterol <40 mg/dL, triglycerides ≥150 mg/dL, or use of lipid-lowering medication

We compared individuals with (*n* = 63,785) and without (*n* = 33,175) follow-up health check-up data at 1 year after their first check-up (Supplementary Table 1). The proportion of men was significantly lower in individuals without follow-up data. The proportion of individuals who visited a physician within 3 months of their health check-up was slightly lower in those without follow-up data (53.1%) compared to those with follow-up data (54.5%, *P* < 0.001). However, the rate of antihypertensive prescriptions within 3 months was nearly identical between the two groups (16.5% vs. 16.6%, *P* = 0.60). To further explore the relationship between follow-up BP, medical visits, and antihypertensive treatment status, we categorized participants into six groups based on their follow-up status and provided their characteristics in Supplementary Table 2.

Factors associated with having grade II or severer hypertension at 1 year after the first check-up were identified as per 5 years lower in age (RR: 1.01, 95% CI: 1.01–1.02), obesity (RR: 1.04, 95% CI: 1.02–1.06), per 5 mmHg higher in SBP (RR: 1.05, 95% CI: 1.04–1.05), per 5 mmHg higher in DBP (RR: 1.07, 95% CI: 1.06–1.07), and skipping breakfast ≥3 times per week (RR: 1.06, 95% CI: 1.04–1.08) (Table 3). In the analysis adjusted for the visit to a medical institution within 3 months, the visit to a physician within 3 months after undergoing a health check-up was associated with a reduced risk of SBP ≥ 160 mmHg or DBP ≥ 100 mmHg at 1 year after the first check-up (Table 4).

**Table 3:** Factors associated with having grade II or severer hypertension at 1 year after the first check-up

	Overall (*n* = 63,785)	
	RR (95% CI)	*P*-value
Per 5 years lower in age	1.01 (1.01–1.02)	<0.001
Sex, men	1.01 (0.99–1.04)	0.188
Obesity	1.04 (1.02–1.06)	<0.001
Per 5 mmHg higher in systolic blood pressure	1.05 (1.04–1.05)	<0.001
Per 5 mmHg higher in diastolic blood pressure	1.07 (1.06–1.07)	<0.001
Diabetes mellitus	0.98 (0.95–1.01)	0.106
Dyslipidemia	0.98 (0.97–1.00)	0.053
Cigarette smoking	1.02 (1.00–1.04)	0.096
Alcohol consumption	1.00 (0.98–1.02)	0.848
Physical inactivity	0.99 (0.97–1.01)	0.206
Poor sleep quality	0.99 (0.98–1.01)	0.510
Skipping breakfast ≥3 times per week	1.06 (1.04–1.08)	<0.001

*P*-values were calculated by the Poisson regression with robust error variance analyses. Obesity was defined as body mass index ≥25 kg/m^2^. Diabetes was defined as fasting glucose ≥126 mg/dL or use of glucose-lowering medications. Dyslipidemia was defined as low-density lipoprotein cholesterol ≥140 mg/dL, high-density lipoprotein cholesterol <40 mg/dL, triglycerides ≥150 mg/dL, or use of lipid-lowering medications. All variables are simultaneously included in the model

*RR* relative risk, *CI* confidence interval

**Table 4:** Factors associated with having grade II or severer hypertension at 1 year after the first check-up adjusted for the visit to a physician within 3 months after undergoing a health checkup

	Overall (*n* = 63,785)	
	RR (95% CI)	*P*-value
Per 5 years lower in age	1.01 (1.00–1.01)	0.002
Sex, men	0.98 (0.96–1.01)	0.156
Obesity	1.04 (1.02–1.06)	<0.001
Per 5 mmHg higher in systolic blood pressure	1.05 (1.04–1.05)	<0.001
Per 5 mmHg higher in diastolic blood pressure	1.07 (1.06–1.07)	<0.001
Diabetes mellitus	0.99 (0.97–1.02)	0.698
Dyslipidemia	0.99 (0.97–1.00)	0.129
Cigarette smoking	0.99 (0.97–1.01)	0.473
Alcohol consumption	1.00 (0.98–1.02)	0.985
Physical inactivity	0.99 (0.97–1.01)	0.253
Poor sleep quality	1.00 (0.99–1.02)	0.799
Skipping breakfast ≥3 times per week	1.03 (1.01–1.05)	0.002
No physician visit in 3 months after undergoing a health checkup	1.36 (1.34–1.38)	<0.001

*P*-values were calculated using the Poisson regression with robust error variance analyses adjusted for a variable of the visit to a physician within 3 months after undergoing a health checkup. Obesity was defined as body mass index ≥25 kg/m^2^. Diabetes was defined as fasting glucose ≥126 mg/dL or use of glucose-lowering medications. Dyslipidemia was defined as low-density lipoprotein cholesterol ≥140 mg/dL, high-density lipoprotein cholesterol <40 mg/dL, triglycerides ≥150 mg/dL, or use of lipid-lowering medications. All variables are simultaneously included in the model

*RR* relative risk, *CI* confidence interval

Factors associated with not receiving antihypertensive medications at 1 year after the first check-up were identified as per 5 years lower in age (RR: 1.02, 95% CI: 1.02–1.02), and skipping breakfast ≥3 times per week (RR: 1.05, 95% CI: 1.04–1.06) (Table 5). Consistent associations were observed across age category and sex (Supplementary Tables 3 and 4). Supplementary Table 5 presented the factors associated with having grade II or severer hypertension at 1 year after the first check-up, stratified by BMI categories at baseline. In the BMI < 25 group, no significant association was observed between age and grade II or severer hypertension, whereas in the BMI ≥ 25 group, lower age was associated with a higher risk (RR: 1.02, 95% CI: 1.02–1.03). Skipping breakfast ≥3 times per week was associated with an increased risk in both BMI groups. Supplementary Table 6 was shown the factors associated with not receiving antihypertensive medications at 1 year after the first check-up, stratified by BMI category. Regardless of BMI, higher BP levels were associated with a lower proportion of individuals remaining untreated. Also, skipping breakfast was associated with a higher proportion of individuals not receiving antihypertensive treatment in both BMI groups. In addition, the analysis was conducted specifically among individuals aged 40–64 years (Supplementary Tables 7–9), the results were generally consistent with those in the overall analysis.

**Table 5:** Factors associated with not receiving antihypertensive medications at 1 year after the first check-up

	Overall (*n* = 63,785)	
	RR (95% CI)	*P*-value
Per 5 years lower in age	1.02 (1.02–1.02)	<0.001
Sex, men	1.00 (0.99–1.01)	0.687
Obesity	0.97 (0.96–0.98)	<0.001
Per 5 mmHg higher in systolic blood pressure	0.98 (0.97–0.98)	<0.001
Per 5 mmHg higher in diastolic blood pressure	0.96 (0.96–0.97)	<0.001
Diabetes mellitus	1.00 (0.99–1.02)	0.668
Dyslipidemia	1.00 (0.99–1.01)	0.672
Cigarette smoking	0.99 (0.98–1.00)	0.092
Alcohol consumption	0.99 (0.98–1.00)	0.087
Physical inactivity	0.97 (0.97–0.98)	<0.001
Poor sleep quality	0.97 (0.96–0.98)	<0.001
Skipping breakfast ≥3 times per week	1.05 (1.04–1.06)	<0.001

*P*-values were calculated by the Poisson regression with robust error variance analyses. We obtained the medication prescriptions for hyperglycemia (WHO-ATC codes starting with A10) and dyslipidemia (WHO-ATC codes starting with C10) from the claims data after the health checkup. Obesity was defined as body mass index ≥25 kg/m^2^. Diabetes was defined as fasting glucose ≥126 mg/dL or use of glucose-lowering medications. Dyslipidemia was defined as low-density lipoprotein cholesterol ≥140 mg/dL, high-density lipoprotein cholesterol <40 mg/dL, triglycerides ≥150 mg/dL, or use of lipid-lowering medications. All variables are simultaneously included in the model

*RR* relative risk, *CI* confidence interval

## Discussion

In our analysis using a large-scale database in Japan, we found that among individuals in the health check-up with SBP ≥ 160 mmHg or DBP ≥ 100 mmHg, who were strongly recommended for medication consultation, 45.4% individuals had SBP ≥ 160 mmHg or DBP ≥ 100 mmHg at 1 year after the first check-up. 54.5% individuals visited a medical institution within 3 months after undergoing a health check-up, and 23.6% were prescribed antihypertensive medications at 1 year after the first check-up. Factors associated with grade II or severer hypertension (SBP ≥ 160 mmHg or DBP ≥ 100 mmHg) at 1 year after the first check-up included younger age, obesity, and skipping breakfast ≥3 times per week. To the best of our knowledge, this is the first large-scale epidemiological analysis to evaluate BP control after the first check-up among individuals with SBP ≥ 160 mmHg or DBP ≥ 100 mmHg, a level that requires immediate medical consultation according to the health check-ups in Japan.

Hypertension has a high global prevalence and is the leading preventable CVD risk factor worldwide. However, despite the availability of a range of pharmacological treatment options, BP control is often suboptimal [4, 16–19]. In Japan, a steady decrease in BP levels was observed over a 55-year period. The prevalence of hypertension remains high: over 60% of men aged ≥50 years and women aged ≥60 years display hypertension. However, the control rates of hypertension have shown continuous improvement: they have increased to ~40% over a 36-year period. Nonetheless, the over 50% prevalence of uncontrolled hypertension is a considerable risk for future CVD [7]. Satoh et al. [19] analyzed 27,652 hypertensive patients, showing that 43% had uncontrolled BP (SBP ≥ 140 mmHg or DBP ≥ 90 mmHg) after receiving antihypertensive treatment in Japan. Inadequate treatment, particularly with fewer than three antihypertensive drugs, was a major factor for uncontrolled BP. Consistent with preceding studies, our investigation also highlights the clinical challenges of BP management in real-world clinical settings. Several reviews have emphasized the importance of RWD in understanding and addressing the clinical challenges of hypertension management in Japan. Okada [20] highlighted how RWD can provide valuable insights into treatment patterns and outcomes in hypertension management, facilitating more targeted and effective interventions. Similarly, Tonegawa-Kuji et al. [21] reviewed the utilization of RWD in the cardiovascular field, demonstrating its potential to advance disease prevention and management in Japan. These studies support our findings by underscoring the critical role of RWD in identifying gaps in clinical care and informing strategies to improve BP control in real-world settings.

It is recommended that individuals with SBP ≥ 160 mmHg or DBP ≥ 100 mmHg, as identified during the health check-ups, seek medical consultation promptly. However, within our cohort, only 54.5% of individuals with BP in this range visited a medical institution within 3 months after undergoing a health check-up. It should be noted that the actual proportion of individuals seeking care specifically for BP management may be even lower, as these visits might also include follow-ups for other medical conditions. In the analysis adjusted for the visit to a medical institution within 3 months, visiting a medical institution within 3 months after undergoing a health check-up was associated with a reduced risk of SBP ≥ 160 mmHg or DBP ≥ 100 mmHg at 1 year after the first check-up. Adherence to the recommendations of the health check-up and prompt visit to a medical institution is likely to be crucial for effective BP management. Previous studies emphasized how insights from RWD can guide healthcare policies and improve clinical practice, particularly in managing chronic conditions like hypertension [20, 21]. Incorporating these findings, future initiatives in Japan could focus on enhancing follow-up care and intervention programs tailored to specific populations, using RWD to monitor progress and adjust strategies dynamically.

Factors associated with grade II or severer hypertension (SBP ≥ 160 mmHg or DBP ≥ 100 mmHg) at 1 year after the first check-up included younger age, obesity, and skipping breakfast ≥3 times per week. These findings are extremely implicative. In recent years, the importance of BP management in young adults has been strongly recognized epidemiologically and clinically [22–25]. Also, epidemiological studies have demonstrated that elevated BP in young adults is associated with an increased risk of future CVD, with the relative risk for young adults being higher than that for older individuals [26–29]. On the other hand, considering that young adults have a low awareness of health [30], the findings of our study also suggest such a possibility. Therefore, appropriate health education initiatives and social support are urgently needed for this population.

Additionally, obesity itself is a risk factor for hypertension, and the coexistence of obesity and hypertension further increases the risk of future CVD [25, 31, 32]. It should also be emphasized that obesity is associated with a higher prevalence of diabetes and dyslipidemia [33]. For hypertensive cases with obesity detected during health screening, stronger recommendations for medical consultation and appropriate referral to healthcare facilities are needed, as the current study showed that obesity was associated with a higher risk of grade II or severer hypertension. Furthermore, unhealthy lifestyle habits, such as skipping breakfast, are known to be linked to increased risks of hypertension and future CVD in previous studies [34–38]. However, it remains unclear whether skipping breakfast directly contributes to elevated BP or if the underlying irregular lifestyle or work conditions (e.g., shift work) associated with skipping breakfast are related to uncontrolled BP. Also, lifestyle factors, including alcohol consumption, physical activity, and sleep status, differed depending on treatment status in the current study. Further investigation is needed to clarify the relationship between these lifestyle factors and hypertension treatment status.

The strength of this study was to evaluate the outcomes of individuals with referral-level hypertension at 1 year after the first check-up, using claims data in Japan, which highlights significant gaps in strong recommendations for medical consultations and treatment implementation. However, our study has several limitations. First, the study was based on the observational design and data from Japanese health check-up and claims database, which may limit the generalizability of the findings. The challenges of using RWD for research have been discussed in previous studies. Satoh et al. [39] noted that while RWD provides unique insights into hypertension and its association with cardiovascular or renal diseases, issues such as data quality, missing information, and potential biases need careful consideration. These limitations align with those in our study, including the difficulty in strictly differentiating secondary hypertension from the data, as well as unmeasured confounders and selection biases inherent in claims-based databases. Addressing these challenges will be crucial for future research leveraging RWD to produce more robust and generalizable findings. Second, the variables such as socioeconomic status are not included in the database, it is not possible to completely rule out the potential for residual unmeasured confounders. Nevertheless, given that Japan provides universal health coverage to all its citizens, economic factors are less likely to significantly influence the decision-making process for initiating treatment with hypertension drugs. Third, it was possible that selection bias occurred during the process of including and excluding participants. However, when comparing individuals with and without follow-up health check-up data at 1 year, the rate of antihypertensive prescriptions within 3 months was nearly identical between the two groups in the current study. Fourth, the use of antihypertensive medications might have been overreported in the claims database due to the potential misclassification of drugs coded under WHO-ATC categories. While these codes are commonly associated with antihypertensive drugs, they may also include medications prescribed for other conditions, such as heart failure or arrhythmias. To address this issue, we focused on patients with documented BP values indicative of hypertension (grade II or higher) to minimize the impact of non-hypertensive prescriptions. However, it is important to acknowledge that this approach may not completely eliminate the inclusion of non-hypertensive medications, which could lead to minor inaccuracies in our findings. Fifth, a potential limitation of the current study was the influence of regression to the mean. Individuals with extreme first BP values, such as SBP ≥ 160 mmHg or DBP ≥ 100 mmHg, are more likely to show reductions in subsequent measurements, regardless of any treatment received or lifestyle changes. This statistical phenomenon may have contributed to an underestimation of the actual burden of uncontrolled hypertension among the individuals. Sixth, although the standard BP measurement procedures were recommended as described above, it was not unclear whether these methods were accurately or consistently performed in real-world clinical settings. Finally, to evaluate the long-term effects of BP, longer-term follow-up data are warranted.

### Perspective of Asia

Hypertension remains a major public health issue across Asia, with considerable variation in awareness, treatment, and control rates among countries [40]. Challenges such as limited access to primary care, poor adherence to therapy, and reliance on monotherapy persist. Our study demonstrates a significant treatment gap in the health check-up, particularly among younger, obese individuals or those with unhealthy lifestyles and grade II or more severe hypertension, despite medical recommendations. These findings reflect broader trends across Asia, where hypertension is often detected through screening but not adequately followed by treatment initiation and sustained management. Regionally tailored follow-up strategies and behaviorally informed interventions may help address this gap.

## Conclusion

Our study found that BP control remains inadequate among individuals with grade II or severer hypertension in the health check-up, 45.4% individuals had SBP ≥ 160 mmHg or DBP ≥ 100 mmHg at 1 year after the first check-up despite strong recommendations for medical consultation. It is essential to implement interventions and provide follow-up particularly for younger adults and those with unhealthy lifestyles to enhance BP management.

## Supplementary information

Supplementary Material

## Data Availability

This database is available for anyone who purchases it from JMDC Inc. (https://www.jmdc.co.jp/en/).
