# Supplementary material for: Blood pressure control and treatment status at 1 year after the first health check-up in individuals with observed referral-level blood pressure
Source: Hypertens Res. 2025 Jul 25;48(10):2537–47. doi: 10.1038/s41440-025-02284-y (PMC12497642; doi:10.1038/s41440-025-02284-y)
Supplement: Supplementary file 1 — Supplementary Material [file 41440_2025_2284_MOESM1_ESM.docx]

***Supplementary Material***

Blood pressure control and treatment status at 1 year after the first health check-up in individuals with observed referral-level blood pressure

Kaori Kitaoka, PhD,^1,2^ Hidehiro Kaneko, MD,^2,3^ Yuta Suzuki, PhD,^2,4^ Akira Okada, MD,^5^ Atsushi Mizuno, MD,^6^ Katsuhito Fujiu, MD,^2,3^ Norifumi Takeda, MD,^2^ Hiroyuki Morita, MD,^2^ Tatsuhiko Azegami, MD,^7^ Kaori Hayashi, MD,^7^ Koichi Node, MD,^8^ Yuji Furui, PhD,^9^ Katsuyuki Miura, MD,^1,10^ Hideo Yasunaga, MD,^11^ Norihiko Takeda, MD. ^2^

1. NCD Epidemiology Research Center, Shiga University of Medical Science, Otsu, Japan
2. Department of Cardiovascular Medicine, The University of Tokyo, Tokyo, Japan
3. Department of Advanced Cardiology, The University of Tokyo, Tokyo, Japan.
4. Center for Outcomes Research and Economic Evaluation for Health, National Institute of Public Health, Saitama, Japan.
5. Department of Prevention of Diabetes and Lifestyle-Related Diseases, Graduate School of Medicine, The University of Tokyo, Tokyo, Japan.
6. Department of Cardiovascular Medicine, St. Luke's International Hospital, Tokyo, Japan.
7. Division of Nephrology, Endocrinology and Metabolism, Department of Internal Medicine, Keio University School of Medicine, Tokyo, Japan.
8. Department of Cardiovascular Medicine, Saga University, Saga, Japan.
9. Healthcare Data Science Research Unit, Institute for Future Initiatives, The University of Tokyo, Tokyo, Japan
10. Department of Public Health, Shiga University of Medical Science, Otsu, Japan
11. Department of Clinical Epidemiology and Health Economics, School of Public Health, The University of Tokyo, Tokyo, Japan.

Main text: 4,178 words; References: 39; Table: 5; Figures: 1; Supplementary materials:9

Corresponding author:

Hidehiro Kaneko, MD, PhD

Department of Advanced Cardiology, The University of Tokyo

Address: 7-3-1, Hongo, Bunkyo-Ku, Tokyo 113-8655, Japan

E-mail: kanekohidehiro@gmail.com

Tel: +81-3-3815-5411

Supplementary Table 1. Comparison of individuals with and without health check-up data at 1 year after the first check-up

| Variables | Individuals with health check-up data at 1 year after the first check-up (n=63,785) | Individuals without health check-up data at 1 year after the first check-up (n=33,175) | P-value |
| --- | --- | --- | --- |
| Age (years) | 50 (44-56) | 51 (44-58) | <0.001 |
| Sex, men, n (%) | 48,004 (75.3) | 21,019 (63.4) | <0.001 |
| Body mass index (kg/m^2^) | 25.1 (22.6-28.3) | 25.1 (22.3-28.4) | 0.020 |
| Obesity, n (%) | 33,008 (51.7) | 16,950 (51.1) | 0.052 |
| Systolic blood pressure (mmHg) | 160 (149-167) | 162 (152-170) | <0.001 |
| Diastolic blood pressure (mmHg) | 103 (100-108) | 102 (99-108) | <0.001 |
| Diabetes mellitus, n (%) | 5,770 (9.0) | 3,351 (10.1) | <0.001 |
| Dyslipidemia, n (%) | 38,654 (60.6) | 20,017 (60.3) | 0.430 |
| Cigarette smoking, n (%) | 19,917 (31.2) | 10,194 (30.7) | 0.110 |
| Alcohol consumption, n (%) | 23,491 (36.8) | 11,150 (33.6) | <0.001 |
| Physical inactivity, n (%) | 35,523 (55.7) | 17,539 (52.9) | <0.001 |
| Poor sleep quality, n (%) | 24,537 (38.5) | 12,301 (37.1) | <0.001 |
| Skipping breakfast ≥3 times per week, n (%) | 17,687 (27.7) | 9,582 (28.9) | <0.001 |
| Fasting blood glucose (mg/dL) | 98 (91-107) | 98 (91-108) | 0.072 |
| Low-density lipoprotein-cholesterol (mg/dL) | 132 (110-154) | 132 (110-156) | 0.002 |
| High-density lipoprotein-cholesterol(mg/dL) | 58 (48-70) | 59 (49-73) | <0.001 |
| Triglycerides (mg/dL) | 115 (78-173) | 111 (76-167) | <0.001 |
| Visit to a physician within 3 months after undergoing a health checkup, n (%) | 34,789 (54.5) | 17,607 (53.1) | <0.001 |
| Antihypertensive prescriptions within 3 months after undergoing a health checkup, n (%) | 10,596 (16.6) | 5,467 (16.5) | 0.600 |

P values were calculated by the Mann-Whitney U test for continuous variables and chi-square tests for categorical variables. Data are expressed as median (interquartile range) or number (percentage). We obtained the medication prescriptions for hypertension (WHO-ATC codes starting with C02, C03, C04, C07, C08, or C09), dyslipidemia (WHO-ATC codes starting with C10), and hyperglycemia (WHO-ATC codes starting with A10), from the claims data after the health checkup. Obesity was defined as body mass index ≥25 kg/m^2^. Diabetes was defined as fasting glucose ≥126 mg/dL or use of glucose-lowering medications. Dyslipidemia was defined as low-density lipoprotein cholesterol ≥140 mg/dL, high-density lipoprotein cholesterol <40 mg/dL, triglycerides ≥150 mg/dL, or use of lipid-lowering medications.

Supplementary Table 2. Baseline characteristics and treatment status by blood pressure and treatment status category at the one-year follow-up

| Variables | Follow-up BP ≥160/90 mmHg without medical visits (n=14,827) | Follow-up BP <160/90 mmHg without medical visits (n= 10,735) | Follow-up BP ≥160/90 mmHg with medical visits but without antihypertensive treatment (n= 11,452) | Follow-up BP <160/90 mmHg with medical visits but without antihypertensive treatment (n= 11,754) | Follow-up BP ≥160/90 mmHg with medical visits and antihypertensive treatment (n= 2,651) | Follow-up BP <160/90 mmHg with medical visits and antihypertensive treatment (n= 12,366) | P-value |
| --- | --- | --- | --- | --- | --- | --- | --- |
| Age (years) | 49 (43-55) | 49 (43-55) | 49 (44-56) | 50 (44-57) | 50 (44-56) | 51 (45-56) | <0.001 |
| Sex, men, n (%) | 11,887 (80.2) | 8,590 (80.0) | 8,049 (70.3) | 8,273 (70.4) | 1,961 (74.0) | 9,244 (74.8) | <0.001 |
| Body mass index (kg/m^2^) | 25.4 (22.7-28.7) | 24.8 (22.4-27.8) | 25.3 (22.7-28.6) | 24.8 (22.3-27.9) | 25.8 (23.1-29.0) | 25.2 (22.9-28.3) | <0.001 |
| Obesity, n (%) | 7,976 (53.8) | 5,177 (48.2) | 6,086 (53.1) | 5,670 (48.2) | 1,534 (57.9) | 6,565 (53.1) | <0.001 |
| Systolic BP (mmHg) | 161 (152-170) | 155 (146-163) | 160 (151-168) | 155 (145-163) | 167 (157-179) | 162 (152-170) | <0.001 |
| Diastolic BP (mmHg) | 104 (100-109) | 101 (100-104) | 103 (100-108) | 101 (99-104) | 107 (101-114) | 104 (100-109) | <0.001 |
| Diabetes mellitus, n (%) | 1,229 (8.3) | 719 (6.7) | 1,132 (9.9) | 1,235 (10.5) | 310 (11.7) | 1,145 (9.3) | <0.001 |
| Dyslipidemia, n (%) | 9,021 (60.8) | 6,259 (58.3) | 6,909 (60.3) | 7,271 (61.9) | 1,657 (62.5) | 7,537 (60.9) | <0.001 |
| Cigarette smoking, n (%) | 5,379 (36.3) | 3,891 (36.2) | 3,039 (26.5) | 2,901 (24.7) | 871 (32.9) | 3,836 (31.0) | <0.001 |
| Alcohol consumption, n (%) | 5,717 (38.6) | 4,205 (39.2) | 3,936 (34.4) | 4,005 (34.1) | 981 (37.0) | 4,647 (37.6) | <0.001 |
| Physical inactivity, n (%) | 8,178 (55.2) | 5,840 (54.4) | 6,345 (55.4) | 6,459 (55.0) | 1,561 (58.9) | 7,140 (57.7) | <0.001 |
| Poor sleep quality, n (%) | 5,513 (37.2) | 3,789 (35.3) | 4,547 (39.7) | 4,612 (39.2) | 1,100 (41.5) | 4,976 (40.2) | <0.001 |
| Skipping breakfast ≥3 times per week, n (%) | 5,004 (33.7) | 3,398 (31.7) | 2,830 (24.7) | 2,717 (23.1) | 697 (26.3) | 3,041 (24.6) | <0.001 |
| Fasting blood glucose (mg/dL) | 98 (91-107) | 97 (90-106) | 98 (91-108) | 98 (91-107) | 99 (92-109) | 98 (91-107) | <0.001 |
| Low-density lipoprotein-cholesterol (mg/dL) | 133 (111-156) | 131 (109-154) | 131 (110-154) | 131 (109-153) | 132 (110-155) | 132 (110-154) | <0.001 |
| High-density lipoprotein-cholesterol(mg/dL) | 57 (48-69) | 58 (48-70) | 58 (48-70) | 59 (49-72) | 58 (48-70) | 57 (48-69) | <0.001 |
| Triglycerides (mg/dL) | 116 (79-175) | 114 (77-175) | 113 (77-168) | 111 (76-171) | 119 (81-179) | 117 (80-174) | <0.001 |
| Systolic BP at 1 year after the first check-up (mmHg) | 163 (154-174) | 142 (134-150) | 162 (153-171) | 140 (133-149) | 160 (151-168) | 133 (124-141) | <0.001 |
| Diastolic BP at 1 year after the first check-up (mmHg) | 105 (101-111) | 92 (86-96) | 104 (100-110) | 91 (85-95) | 103 (100-108) | 85 (79-91) | <0.001 |

Data are expressed as median (interquartile range) or number (percentage). P values were calculated using the Kruskal-Wallis test for continuous variables and chi-square tests for categorical variables. We obtained the medication prescriptions for hypertension (WHO-ATC codes starting with C02, C03, C04, C07, C08, or C09), dyslipidemia (WHO-ATC codes starting with C10), and hyperglycemia (WHO-ATC codes starting with A10), from the claims data after the health checkup. Obesity was defined as body mass index ≥25 kg/m^2^. Diabetes was defined as fasting glucose ≥126 mg/dL or use of glucose-lowering medications. Dyslipidemia was defined as low-density lipoprotein cholesterol ≥140 mg/dL, high-density lipoprotein cholesterol <40 mg/dL, triglycerides ≥150 mg/dL, or use of lipid-lowering medications.

Abbreviations: BP, blood pressure;

Supplementary Table 3. Factors associated with not receiving antihypertensive medications at 1 year after the first check-up according to age category

|  | Age < 50 years (n=31,364) | | Age ≥ 50 years (n=32,421) | |
| --- | --- | --- | --- | --- |
|  | RR (95% CI) | P-value | RR (95% CI) | P-value |
| Per 5 years lower in age | 1.05 (1.04-1.05) | <0.001 | 1.00 (0.99-1.00) | 0.391 |
| Sex, men | 1.01 (0.99-1.02) | 0.377 | 0.99 (0.97-1.00) | 0.143 |
| Obesity | 1.00 (0.99-1.01) | 0.788 | 0.95 (0.94-0.97) | <0.001 |
| Per 5 mmHg higher in systolic blood pressure | 0.97 (0.97-0.98) | <0.001 | 0.97 (0.97-0.98) | <0.001 |
| Per 5 mmHg higher in diastolic blood pressure | 0.95 (0.95-0.96) | <0.001 | 0.98 (0.97-0.98) | <0.001 |
| Diabetes mellitus | 0.99 (0.96-1.01) | 0.265 | 1.02 (1.00-1.04) | 0.091 |
| Dyslipidemia | 1.00 (0.99-1.01) | 0.982 | 0.99 (0.98-1.01) | 0.265 |
| Cigarette smoking | 1.01 (0.99-1.02) | 0.334 | 0.98 (0.97-1.00) | 0.012 |
| Alcohol consumption | 1.01 (1.00-1.02) | 0.190 | 0.98 (0.97-1.00) | 0.019 |
| Physical inactivity | 0.99 (0.98-1.00) | 0.096 | 0.97 (0.95-0.98) | <0.001 |
| Poor sleep quality | 0.98 (0.97-0.99) | <0.001 | 0.97 (0.96-0.99) | <0.001 |
| Skipping breakfast ≥3 times per week | 1.05 (1.03-1.06) | <0.001 | 1.05 (1.03-1.06) | <0.001 |

P values were calculated by the Poisson regression with robust error variance analyses. We obtained the medication prescriptions for hyperglycemia (WHO-ATC codes starting with A10) and dyslipidemia (WHO-ATC codes starting with C10) from the claims data after the health checkup. Obesity was defined as body mass index ≥25 kg/m^2^. Diabetes was defined as fasting glucose ≥126 mg/dL or use of glucose-lowering medications. Dyslipidemia was defined as low-density lipoprotein cholesterol ≥140 mg/dL, high-density lipoprotein cholesterol <40 mg/dL, triglycerides ≥150 mg/dL, or use of lipid-lowering medications. All variables are simultaneously included in the model.

Abbreviations: RR, relative risk; CI, confidence interval

Supplementary Table 4. Factors associated with not receiving antihypertensive medications at 1 year after the first check-up according to sex

|  | Men (n=48,004) | | Women (n=15,781) | |
| --- | --- | --- | --- | --- |
|  | RR (95% CI) | P-value | RR (95% CI) | P-value |
| Per 5 years lower in age | 1.02 (1.02-1.03) | <0.001 | 1.01 (1.01-1.02) | <0.001 |
| Obesity | 0.97 (0.96-0.98) | <0.001 | 0.98 (0.96-1.00) | 0.070 |
| Per 5 mmHg higher in systolic blood pressure | 0.98 (0.98-0.98) | <0.001 | 0.97 (0.97-0.97) | <0.001 |
| Per 5 mmHg higher in diastolic blood pressure | 0.96 (0.96-0.96) | <0.001 | 0.97 (0.97-0.98) | <0.001 |
| Diabetes mellitus | 1.00 (0.98-1.02) | 0.934 | 1.02 (0.99-1.06) | 0.229 |
| Dyslipidemia | 0.99 (0.98-1.00) | 0.135 | 1.01 (0.99-1.03) | 0.218 |
| Cigarette smoking | 0.99 (0.98-1.00) | 0.074 | 1.00 (0.97-1.03) | 0.894 |
| Alcohol consumption | 0.99 (0.98-1.00) | 0.216 | 0.99 (0.96-1.01) | 0.279 |
| Physical inactivity | 0.97 (0.96-0.98) | <0.001 | 0.98 (0.97-1.00) | 0.058 |
| Poor sleep quality | 0.97 (0.96-0.98) | <0.001 | 0.97 (0.95-0.99) | 0.002 |
| Skipping breakfast ≥3 times per week | 1.05 (1.04-1.06) | <0.001 | 1.05 (1.03-1.07) | <0.001 |

P values were calculated by the Poisson regression with robust error variance analyses. We obtained the medication prescriptions for hyperglycemia (WHO-ATC codes starting with A10) and dyslipidemia (WHO-ATC codes starting with C10) from the claims data after the health checkup. Obesity was defined as body mass index ≥25 kg/m^2^. Diabetes was defined as fasting glucose ≥126 mg/dL or use of glucose-lowering medications. Dyslipidemia was defined as low-density lipoprotein cholesterol ≥140 mg/dL, high-density lipoprotein cholesterol <40 mg/dL, triglycerides ≥150 mg/dL, or use of lipid-lowering medications. All variables are simultaneously included in the model.

Abbreviations: RR, relative risk; CI, confidence interval

Supplementary Table 5. Factors associated with having grade II or severer hypertension at 1 year after the first check-up according to BMI category at the baseline

|  | BMI <25 (n=30,777) | | BMI ≥25 (n=33,008) | |
| --- | --- | --- | --- | --- |
|  | RR (95% CI) | P-value | RR (95% CI) | P-value |
| Per 5 years lower in age | 1.00 (1.00-1.01) | 0.403 | 1.02 (1.02-1.03) | <0.001 |
| Sex, men | 1.03 (1.00-1.06) | 0.066 | 0.99 (0.96-1.02) | 0.656 |
| Per 5 mmHg higher in systolic blood pressure | 1.05 (1.05-1.06) | <0.001 | 1.04 (1.04-1.04) | <0.001 |
| Per 5 mmHg higher in diastolic blood pressure | 1.08 (1.07-1.08) | <0.001 | 1.06 (1.06-1.07) | <0.001 |
| Diabetes mellitus | 1.01 (0.96-1.06) | 0.677 | 0.96 (0.93-1.00) | 0.033 |
| Dyslipidemia | 0.97 (0.95-1.00) | 0.041 | 0.99 (0.96-1.01) | 0.343 |
| Cigarette smoking | 1.00 (0.97-1.03) | 0.972 | 1.02 (1.00-1.05) | 0.051 |
| Alcohol consumption | 1.02 (0.99-1.04) | 0.208 | 0.99 (0.96-1.01) | 0.335 |
| Physical inactivity | 0.99 (0.96-1.01) | 0.275 | 0.99 (0.97-1.02) | 0.509 |
| Poor sleep quality | 1.00 (0.97-1.02) | 0.832 | 0.99 (0.97-1.02) | 0.561 |
| Skipping breakfast ≥3 times per week | 1.05 (1.02-1.08) | 0.001 | 1.06 (1.03-1.09) | <0.001 |

P values were calculated by the Poisson regression with robust error variance analyses. We obtained the medication prescriptions for hyperglycemia (WHO-ATC codes starting with A10) and dyslipidemia (WHO-ATC codes starting with C10) from the claims data after the health checkup. Diabetes was defined as fasting glucose ≥126 mg/dL or use of glucose-lowering medications. Dyslipidemia was defined as low-density lipoprotein cholesterol ≥140 mg/dL, high-density lipoprotein cholesterol <40 mg/dL, triglycerides ≥150 mg/dL, or use of lipid-lowering medications. All variables are simultaneously included in the model.

Abbreviations: RR, relative risk; CI, confidence interval

Supplementary Table 6. Factors associated with not receiving antihypertensive medications at 1 year after the first check-up according to BMI category at the baseline

|  | BMI <25 (n=30,777) | | BMI ≥25 (n=33,008) | |
| --- | --- | --- | --- | --- |
|  | RR (95% CI) | P-value | RR (95% CI) | P-value |
| Per 5 years lower in age | 1.01 (1.01-1.01) | <0.001 | 1.03 (1.03-1.04) | <0.001 |
| Sex, men | 1.01 (0.99-1.02) | 0.357 | 0.99 (0.98-1.01) | 0.480 |
| Per 5 mmHg higher in systolic blood pressure | 0.98 (0.97-0.98) | <0.001 | 0.97 (0.97-0.98) | <0.001 |
| Per 5 mmHg higher in diastolic blood pressure | 0.97 (0.97-0.97) | <0.001 | 0.96 (0.95-0.96) | <0.001 |
| Diabetes mellitus | 1.01 (0.98-1.03) | 0.687 | 1.00 (0.98-1.02) | 0.800 |
| Dyslipidemia | 0.99 (0.98-1.00) | 0.188 | 1.00 (0.99-1.01) | 0.836 |
| Cigarette smoking | 0.99 (0.98-1.01) | 0.380 | 0.99 (0.98-1.00) | 0.145 |
| Alcohol consumption | 0.99 (0.98-1.01) | 0.398 | 0.99 (0.98-1.01) | 0.214 |
| Physical inactivity | 0.97 (0.96-0.98) | <0.001 | 0.98 (0.97-0.99) | <0.001 |
| Poor sleep quality | 0.97 (0.96-0.99) | <0.001 | 0.97 (0.96-0.99) | <0.001 |
| Skipping breakfast ≥3 times per week | 1.04 (1.02-1.05) | <0.001 | 1.05 (1.04-1.07) | <0.001 |

P values were calculated by the Poisson regression with robust error variance analyses. We obtained the medication prescriptions for hyperglycemia (WHO-ATC codes starting with A10) and dyslipidemia (WHO-ATC codes starting with C10) from the claims data after the health checkup. Diabetes was defined as fasting glucose ≥126 mg/dL or use of glucose-lowering medications. Dyslipidemia was defined as low-density lipoprotein cholesterol ≥140 mg/dL, high-density lipoprotein cholesterol <40 mg/dL, triglycerides ≥150 mg/dL, or use of lipid-lowering medications. All variables are simultaneously included in the model.

Abbreviations: RR, relative risk; CI, confidence interval

Supplementary Table 7. Baseline characteristics and treatment status of individuals aged 40-64 years

| Variables | Individuals aged 40-64 years (n=54,476) |
| --- | --- |
| Age (years) | 50 (46-56) |
| Sex, men, n (%) | 40,811 (74.9) |
| Body mass index (kg/m^2^) | 25.0 (22.6-28.0) |
| Obesity, n (%) | 27,625 (50.7) |
| Systolic blood pressure (mmHg) | 160 (149-167) |
| Diastolic blood pressure (mmHg) | 103 (100-108) |
| Diabetes mellitus, n (%) | 4,918 (9.0) |
| Dyslipidemia, n (%) | 32,993 (60.6) |
| Cigarette smoking, n (%) | 17,140 (31.5) |
| Alcohol consumption, n (%) | 20,829 (38.2) |
| Physical inactivity, n (%) | 30,915 (56.7) |
| Poor sleep quality, n (%) | 21,358 (39.2) |
| Skipping breakfast ≥3 times per week, n (%) | 14,611 (26.8) |
| Fasting blood glucose (mg/dL) | 98 (91-107) |
| Low-density lipoprotein-cholesterol (mg/dL) | 132 (110-155) |
| High-density lipoprotein-cholesterol(mg/dL) | 58 (49-71) |
| Triglycerides (mg/dL) | 114 (78-173) |
| Visit to a physician within 3 months after undergoing a health checkup, n (%) | 29,796 (54.7) |
| Antihypertensive prescriptions within 3 months after undergoing a health checkup, n (%) | 9,361 (17.2) |
| Antihypertensive prescriptions at 1 year after the first check-up, n (%) | 13,379 (24.6) |
| Systolic blood pressure at 1 year after the first check-up (mmHg) | 148 (136-161) |
| Diastolic blood pressure at 1 year after the first check-up (mmHg) | 96 (88-104) |

Data are expressed as median (interquartile range) or number (percentage). We obtained the medication prescriptions for hypertension (WHO-ATC codes starting with C02, C03, C04, C07, C08, or C09), dyslipidemia (WHO-ATC codes starting with C10), and hyperglycemia (WHO-ATC codes starting with A10), from the claims data after the health checkup. Obesity was defined as body mass index ≥25 kg/m^2^. Diabetes was defined as fasting glucose ≥126 mg/dL or use of glucose-lowering medications. Dyslipidemia was defined as low-density lipoprotein cholesterol ≥140 mg/dL, high-density lipoprotein cholesterol <40 mg/dL, triglycerides ≥150 mg/dL, or use of lipid-lowering medications.

Supplementary Table 8. Factors associated with having grade II or severer hypertension at 1 year after the first check-up among individuals aged 40-64 years

|  | Individuals aged 40-64 years (n=54,476) | |
| --- | --- | --- |
|  | RR (95% CI) | P-value |
| Per 5 years lower in age | 1.02 (1.01-1.03) | <0.001 |
| Sex, men | 1.02 (1.00-1.05) | 0.070 |
| Obesity | 1.03 (1.01-1.05) | 0.001 |
| Per 5 mmHg higher in systolic blood pressure | 1.05 (1.04-1.05) | <0.001 |
| Per 5 mmHg higher in diastolic blood pressure | 1.06 (1.06-1.07) | <0.001 |
| Diabetes mellitus | 0.97 (0.94-1.01) | 0.112 |
| Dyslipidemia | 0.98 (0.96-1.00) | 0.015 |
| Cigarette smoking | 1.01 (0.99-1.03) | 0.224 |
| Alcohol consumption | 1.00 (0.99-1.03) | 0.624 |
| Physical inactivity | 0.99 (0.97-1.01) | 0.341 |
| Poor sleep quality | 0.99 (0.97-1.01) | 0.291 |
| Skipping breakfast ≥3 times per week | 1.05 (1.03-1.08) | <0.001 |

P values were calculated by the Poisson regression with robust error variance analyses. We obtained the medication prescriptions for hyperglycemia (WHO-ATC codes starting with A10) and dyslipidemia (WHO-ATC codes starting with C10) from the claims data after the health checkup. Obesity was defined as body mass index ≥25 kg/m^2^. Diabetes was defined as fasting glucose ≥126 mg/dL or use of glucose-lowering medications. Dyslipidemia was defined as low-density lipoprotein cholesterol ≥140 mg/dL, high-density lipoprotein cholesterol <40 mg/dL, triglycerides ≥150 mg/dL, or use of lipid-lowering medications. All variables are simultaneously included in the model.

Abbreviations: RR, relative risk; CI, confidence interval

Supplementary Table 9. Factors associated with not receiving antihypertensive medications at 1 year after the first check-up among individuals aged 40-64 years

|  | Individuals aged 40-64 years (n=54,476) | |
| --- | --- | --- |
|  | RR (95% CI) | P-value |
| Per 5 years lower in age | 1.01 (1.01-1.02) | <0.001 |
| Sex, men | 1.00 (0.99-1.01) | 0.635 |
| Obesity | 0.97 (0.96-0.98) | <0.001 |
| Per 5 mmHg higher in systolic blood pressure | 0.97 (0.97-0.98) | <0.001 |
| Per 5 mmHg higher in diastolic blood pressure | 0.96 (0.96-0.97) | <0.001 |
| Diabetes mellitus | 1.01 (0.99-1.02) | 0.550 |
| Dyslipidemia | 1.00 (0.99-1.01) | 0.679 |
| Cigarette smoking | 0.99 (0.98-1.00) | 0.105 |
| Alcohol consumption | 0.99 (0.98-1.00) | 0.293 |
| Physical inactivity | 0.97 (0.96-0.98) | <0.001 |
| Poor sleep quality | 0.97 (0.96-0.98) | <0.001 |
| Skipping breakfast ≥3 times per week | 1.05 (1.03-1.06) | <0.001 |

P values were calculated by the Poisson regression with robust error variance analyses. We obtained the medication prescriptions for hyperglycemia (WHO-ATC codes starting with A10) and dyslipidemia (WHO-ATC codes starting with C10) from the claims data after the health checkup. Obesity was defined as body mass index ≥25 kg/m^2^. Diabetes was defined as fasting glucose ≥126 mg/dL or use of glucose-lowering medications. Dyslipidemia was defined as low-density lipoprotein cholesterol ≥140 mg/dL, high-density lipoprotein cholesterol <40 mg/dL, triglycerides ≥150 mg/dL, or use of lipid-lowering medications. All variables are simultaneously included in the model.

Abbreviations: RR, relative risk; CI, confidence interval
